# Single cell RNA-Sequencing Reveals Mast Cells Enhance Mononuclear Phagocytes Infiltration in Bladder Cancer Microenvironment

**DOI:** 10.7150/jca.99554

**Published:** 2024-09-03

**Authors:** Zige Liu, Caisheng Huang, Xingning Mao, Junhao Mi, Qingyun Zhang, Yuli Xie, Hao Yuan, Mujia Jili, Jiange Zhang, Jianxin Chen, Shengzhu Huang, Zengnan Mo, Rirong Yang

**Affiliations:** 1 Institute of Urology and Nephrology, First Affiliated Hospital of Guangxi Medical University, Guangxi Medical University, Nanning 530021, Guangxi, China.; 2 Center for Genomic and Personalized Medicine, Guangxi key Laboratory for Genomic and Personalized Medicine, Guangxi Collaborative Innovation Center for Genomic and Personalized Medicine, Guangxi Medical University, Nanning 530021, Guangxi, China.; 3 Department of Urology, The Nanning Second People's Hospital, The Third Affiliated Hospital of Guangxi Medical University, Nanning 530021, Guangxi, China.; 4 Department of Urology, The Affiliated Tumor Hospital of Guangxi Medical University, Nanning 530021, Guangxi, China.; 5 Department of Immunology, School of Basic Medical Sciences, Guangxi Medical University, Nanning 530021, Guangxi, China.; 6 Department of Urology, The Second Affiliated Hospital of Guangxi Medical University, Nanning 530021, Guangxi, China.

**Keywords:** Blader Cancer, Mast Cell, Mononuclear Phagocytes, Single Cell RNA-Sequencing, Tumor Microenvironment

## Abstract

**Objective:** Investigating the interaction between Mast cells (MCs) and Mononuclear Phagocytes (MPs) in the tumor microenvironment (TME) of blader cancer (BCa) to uncover potential immunotherapeutic targets.

**Methods:** Single-cell RNA sequencing (scRNA-Seq) was conducted on 12 BCa patients to identify distinct subgroups of MCs and MPs. Transcriptome data was analyzed to characterize the phenotype, gene enrichment, cell-cell communication, and biological processes. The expression levels of cytokines were assessed by enzyme-linked immunosorbent assay (ELISA), while the chemotactic effects of cytokines were evaluated through Transwell assay.

**Results:** In muscle-invasive bladder cancer (MIBC), the proportion of interferon-stimulated MC subtype (Mast-ISG15) increased. Mast-IL13 subgroup and Mast-CCL2 subgroups were functionally enriched in interferon (IFN) and nuclear factor kappa-B (NF-κB) signaling pathways. The Mast-CCL2 subgroup overexpressed the *CCL2* gene, which could chemoattract MPs through CCL2. *In vitro* experiments confirmed that under stimulation, activated MCs activated IFN and NF-κB signaling, increasing the secretion of CCL2 and IL-13, chemoattracted THP-1 monocyte.

**Conclusion:** This study revealed the vital role of MCs in shaping the TME of BCa. And provides new insights for the precise treatment of BCa.

## Introduction

BCa is the most common malignant tumor of the genitourinary system, with an incidence second only to prostate cancer among male genitourinary tumors. The incidence of BCa in males is about 3 - 4 times as high as that in females, with a peak occurrence between the ages of 50 and 70^1^. BCa can be classified as non-muscle-invasive bladder cancer (NMIBC) and MIBC, depending on the depth of invasion of urothelial tumor on the bladder wall. In MIBC, the tumor cells infiltrate beyond the bladder mucosa and submucosa, invading the bladder muscle layer, showing local invasion and tumor metastasis characteristics^2^. The treatment of BCa has undergone several revolutions, from surgery to radiotherapy and chemotherapy, to recent immune checkpoint inhibitors, but the overall therapeutic effects are unsatisfactory, with a response rate to immune checkpoint inhibitors of less than 25%^3^. Therefore, further understanding the mechanisms of BCa development is a pressing matter that requires immediate attention.

TME is closely related to the occurrence and development of cancer cells. Therefore, the study of its specific mechanisms can provide new ideas for the treatment of cancer. The analysis of complex interactions of multiple cell types in the TME at the single-cell level provides important information for the development of new therapeutic strategies. MCs are one of the main immune cells in the human body, known for their role as the most direct effector cells causing allergic reactions, but they also participate in many important physiological and pathological processes^4^. MC infiltration has been found in many tumors and plays an important role in the tumor immune microenvironment. The ability of MCs to promote or prevent tumorigenesis has been shown to depend on the tumor type, cancer stage, the activation state of MCs, the location of MCs in the tumor microenvironment, and the balance between tumor-promoting and anti-tumor effects on tumor cells^5^. In bladder cancer tissues, the density of MCs is significantly higher than in normal tissues and is associated with tumor infiltration and expansion^6^. MPs are also an important cell in the body and playing crucial roles in tissue development, maintenance of homeostasis, and modulation of the balance between pro- and anti-inflammatory responses^7^. Due to their inherent heterogeneity, these cells exist in various differentiation states (monocytes, macrophages and dendritic cells) and exhibit diverse activation profiles in response to changing microenvironmental cues. Increasing evidence suggests that MPs play pivotal roles across all stages of cancer progression^8^.

In our previously work, we conducted a single-cell transcriptomic map of the human and mouse bladders^9^. However, the specific types and activation states of MCs within the TME of BCa remain unclear. In this study, we used the method of scRNA-Seq to sequence tumor tissues, adjacent normal tissues, lymph nodes, and peripheral blood collected from NMIBC and MIBC bladder cancer patients, focusing on the functional status analysis of mast cell populations. This systematic approach aims to provide theoretical evidence to elucidate the role of MCs in the MIBC.

## Methods

### Clinical information and pathologic assessment

The study procedures were approved by the Medical Ethics Committee of Guangxi Medical University (Ethical Approval Number: 2018-003). Informed consent was obtained from all participants for sample collection and data analysis. Samples were sourced from the First Affiliated Hospital of Guangxi Medical University and the Affiliated Tumor Hospital of Guangxi Medical University. A total of 12 patients (10 males, 2 females) were included in the study (**Table S1**), with 5 diagnosed with MIBC and 3 with NMIBC. Nonmalignant bladder tissue samples were collected from three patients (P10-P12) as previously described 9. Pathological classification of all BCa patients was performed according to the World Health Organization's guidelines, and none had undergone radiation, chemotherapy, or intracystic treatment prior to surgery 10.

### Tissue dissociation and PBMC isolation

This step has been described in our previous study^9^. Full-thickness samples of the tumor and adjacent nonmalignant tissues were generated with a surface diameter of 1 cm. Briefly, fresh biopsy samples from BCa tissues were cut into small pieces and rinsed with phosphate-buffered saline (PBS; Gibco, Thermo Fisher Scientific, Inc., Waltham, MA, USA). The tissues were then cut into 2-4 mm^2^ fragments using sterile scissors, washed, and resuspended twice. The tissue specimens underwent a 30 min digestion at 37°C in a digestion solution [1 mg/mL collagenase I (Gibco, USA) and 1 mg/mL DNaseI (Roche, Diagnostics, Basel, Switzerland) in HBSS]. The digestion process was terminated with DMEM (WISENT, Bio Products, Canada) containing 10% FBS (Gibco, USA). Subsequently, a 70 μm cell strainer (Falcon, Corning Inc., NY, USA) was used to filter out large tissue fragments. Red blood cells were eliminated using red blood cell lysis buffer (10× diluted to 1×; BioLegend, San Diego, CA, USA) for 5 min on ice. The samples were further filtered through a 40 μm cell strainer (Falcon). Viable cells were counted following trypan blue staining (Gibco), ensuring a cell viability of more than 80% in each sample. Some of the living cells were directly utilized for scRNA-seq, while others were subsequently isolated from single-cell nuclei. The remaining cells were cryopreserved. PBMC from these BCa patients' blood were isolated via density gradient from whole blood using the Lymphocyte Separation Medium processing protocol (Solarbio Science & Technology, Beijing, China). The PBMC layer was collected and washed twice (400 g × 5 min) in incomplete cold DPBS media.

### 10× library preparation and sequencing

The concentration of single-cell suspensions was adjusted to 1,500 cells/µl, and 22,000 cells/sample were loaded onto a Chromium controller using Chromium Single-Cell 3′ Reagent Kits (v3 chemistry). Sequencing was performed on Illumina Nova S6000 instruments.

### Processing of single-cell RNA sequencing (scRNA-seq) data

The scRNA-seq data underwent processing and quantification utilizing the Cell Ranger (3.1.0) pipeline (https://support.10xgenomics.com/single-cell-gene-expression/software/pipelines/latest/using/count) with the '--id --transcriptome --fastqs --localcores' parameters. Initially, the GRCh38 reference for read alignment was sourced from 10× Genomics. The website provided a summary of sample specifics, such as estimated cell count, mean reads per cell, median genes per cell, median unique molecular identifier (UMI) counts per cell, and sequencing saturation. The feature-barcode matrix was converted into a Seurat object using the R package Seurat (version 3.1.1)^11^. Low-quality cells were removed based on specific criteria: mitochondrial gene percentage > 15%, < 300 genes/cell, and > 7,500 genes/cell for tissue samples or > 5,000 genes/cell for PBMC samples. After filtration, the feature-barcode matrix of each sample was normalized using the “NormalizeData” function with default parameters (employing the “LogNormalize” method and scale.factor = 10,000). The top 2,000 highly variable genes (HVFs) were identified using the “FindVariableFeatures” function with the “vst” method. Subsequently, the feature-barcode matrix was scaled using the “ScaleData” function, and doublets for each sample were identified using the R package DoubletFinder, assuming a 5% doublet formation rate^12^.

### Integration of samples, reduction of dimensions, and clustering

Following the exclusion of doublet cells, the refined feature-barcode matrix from all samples was consolidated. To mitigate batch effects among diverse samples, the anchor correspondences of the combined data were identified using the “FindIntegrationAnchors” function, utilizing the initial 50 dimensions for computation. Subsequently, these established anchors were employed to integrate the combined data through the “IntegrateData” function. The integrated data underwent normalization, followed by the identification of highly variable genes (HVFs) in Seurat. The variables “percent.mito” and “nCount_RNA” were removed through regression using the “ScaleData” function, followed by principal component analysis (PCA) conducted via the “RunPCA” function. The top 50 principal components were employed for dimensionality reduction to visualize cells using the “RunTSNE” function. Primary clusters were identified using the “FindNeighbors” and “FindClusters” functions, with a resolution of 1.4 for optimal clustering. Notably, cells expressing genes associated with multiple lineages were excluded from subsequent analyses.

### Identification of marker genes for clusters

Differential gene expression analysis was conducted using the Seurat function “FindAllMarkers.” Marker genes for major clusters or cell subtypes present in at least 25% of cells were arranged based on their mean log2 (fold change) and filtered with a minimum log2 (fold change) threshold of 0.25. A gene was deemed significantly different if the adjusted *p* < 0.05 (*adj.p*). The top 50 differentially expressed genes within each major cluster or subtype were scrutinized for enrichment in biological processes using Metascape^13^.

### Single-Cell Regulatory Network Inference and Clustering (SCENIC) analysis

To unravel the regulatory landscape of cell clusters, SCENIC was harnessed to dissect gene regulatory networks, following established methodologies^14^. Initially, co-expression modules were inferred using the "runGENIE3" function, and potential direct binding targets (regulons) of transcription factors were pinpointed utilizing the human motif database within a 10 kb radius around the transcription start site (TSS) via RcisTarget. Subsequently, the activity of regulons within cell clusters was assessed through AUCell and averaged. Heatmaps for each regulon cluster were generated using the R package pheatmap.

### Exploration of RNA velocity dynamics

For the analysis of RNA velocity, the velocyto.R tool was utilized^15^. Initially, spliced and unspliced reads for each sample were annotated using velocyto.py with the possorted_genome_bam.bam file, which was generated by Cell Ranger and subsequently stored in a .loom file. Subsequently, the .loom files for each sample were imported into R and merged to create count tables containing both spliced and unspliced reads. Cells accounting for the bottom 0.5% of the total unspliced transcript count were excluded. Genes exhibiting an average expression of spliced variants below 0.2 or unspliced variants below 0.05 in at least one cluster were eliminated. Ultimately, arrows denoting RNA velocity information were integrated into the tSNE plot obtained from Seurat.

### Analysis of cell-cell interactions

The evaluation of cell-cell interactions involved the computation of Ligand-Receptor (L-R) interaction scores utilizing the R package CellChat^16^. Interaction scores between different cell subtypes, such as MC and MP, were calculated and presented for relevant L-R pairs.

### Enzyme linked immunosorbent assay (ELISA)

The THP-1 cell lines were grown in Roswell Park Memorial Institute 1640 (RPMI, Gibco) supplemented with 10% heat-inactivated fetal bovine serum (FBS, Gibco) and 1% penicillin/streptomycin. All cells were incubated in incubators (5% CO_2_ and 37 ℃) and used in subsequent experiments when the cells were in a logarithmic phase of growth. An ELISA kit was used to measure the level of IL-13 (Mlbio, Shanghai, China) and CCL2 (Mlbio) expression in the cell culture supernatant based on the manufacturer's protocol. Optical density (OD) values were read at 450 nm using an enzyme labeler (BioTek, USA).

### Chemotaxis assays

Chemotaxis assays were performed using 24-well transwell plates with 5 µm pores (Corning), following the guidelines provided by the manufacturer. Recombinant human CCL2 (10μg/mL, MCE, Monmouth Junction, USA) was added to the bottom wells. THP-1 (1.5×10^5^) were allowed to migrate for 15 hours at 37 °C in 5% CO_2_. Migrating cells from the lower chamber were collected and counted.

### Cell viability assay

To verify whether increasing serum concentration and CCL2 cytokine levels promote the proliferation of THP-1, a cell viability assay was conducted using cell counting kit-8 (CCK-8, KeyGen, Jiangsu, China). THP-1 cells (5×10^3^ cells/well) were seeded in a 96-well plate, treated with specified concentrations of FBS (2%, and 10%) for 24 hours. Subsequently, 10 μL of CCK-8 assay solution was added to each well, and the cells were further incubated for 3 hours. Absorbance was measured at 450 nm using a microplate reader (BioTek).

## Results

### Single-cell sequencing and cell type identification, quality control of MCs

**Figure 1:**
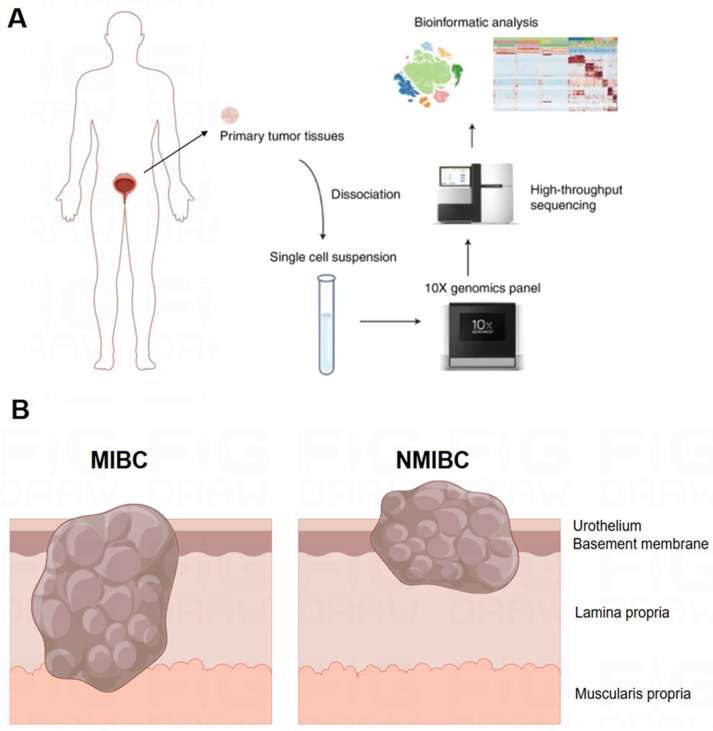
** Workflow and Sampling Schematic of Single-cell RNA Sequencing.** (A) Schematic of the workflow. (B) Schematic diagram showing full-thickness samples of the tumor and adjacent nonmalignant tissues were generated.

**Figure 2:**
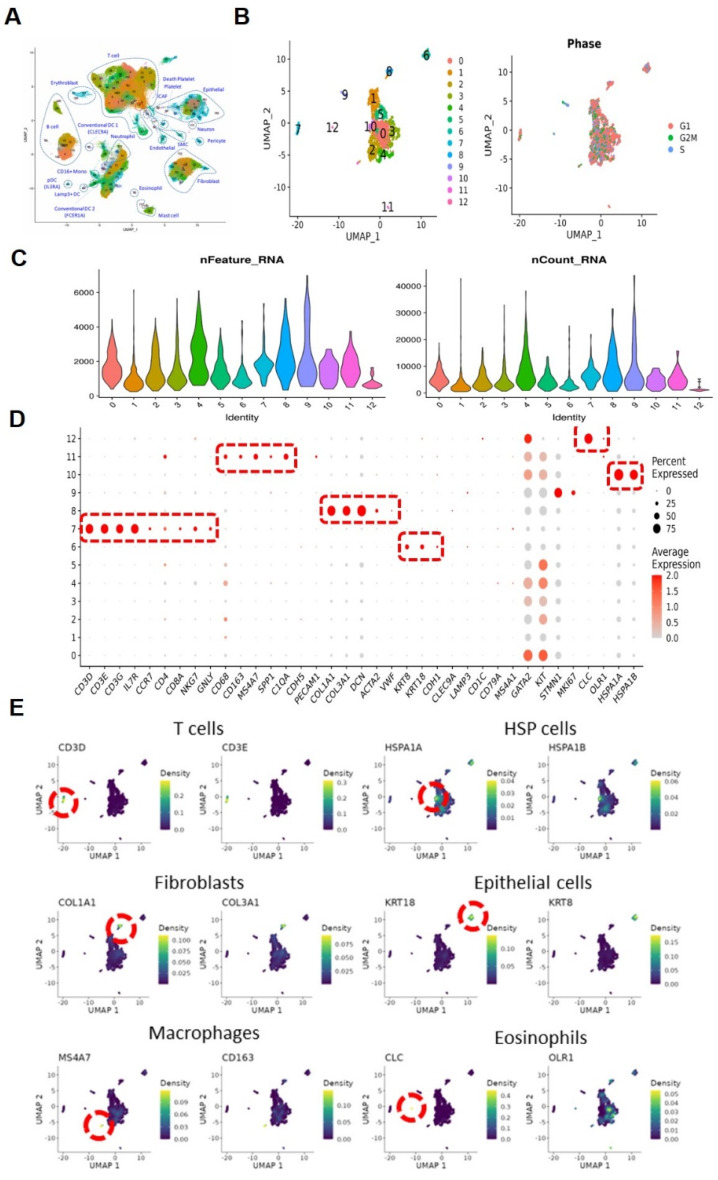
** Single-cell sequencing and cell type identification, quality control of Mast cells.** (A) The general view of bladder cancer in single-cell level. (B) The UMAP graph of Mast cells before quality control. (C) The violin plots show gene numbers (nFeatures) and RNA transcripts (nCounts) in each subgroup. (D) Bubble plot displays the expression of functional markers for each cell subtype. (E) The UMAP plots show the expression levels of cell type marker genes.

As illustrated in the flowchart (**Figure 1A**), this section of the study collected a total of 19 tissue samples from 12 patients. The samples included 10 tumor samples (7 MIBC tumor samples, 3 NMIBC tumor samples) and 9 adjacent normal tissue samples (**Figure 1B**). A total of 179,769 cells were analyzed, including 55,721 single cells from MIBC samples, 19,887 single cells from NMIBC samples, PBMC 33,816, and LNT 18,562. We integrated these scRNA-seq data into a single dataset. We first obtained the single-cell atlas of bladder cancer (**Figure 2A**), with major cell types including epithelial cells, fibroblasts, T cells, B cells, MCs, MPs, dendritic cells, etc. Next, we reclustered the subgroups of MCs for analysis, and MCs were clustered into 13 cell clusters (**Figure 2B**). The first step is to remove contaminating cells and low-quality cells. It can be seen that the feature RNA and RNA quantity of cluster 12 are both below the threshold, indicating that this subgroup of cells is of low quality and should be excluded from subsequent analysis of MC clusters (**Figure 2C**). To further eliminate contaminating cells, we used feature genes for labeling, and plotted bubble charts and gene expression density maps to show the expression levels of characteristic genes of each cell cluster (**Figure 2D**). The bubble chart results show that cluster 6 cells highly express epithelial cell characteristic genes *KRT8* and *KRT18*, cluster 7 cells highly express T cell characteristic genes *CD3D* and *CD3E*, cluster 8 cells highly express fibroblast characteristic genes *COL1A1* and *COL3A1*, cluster 10 cells highly express heat shock proteins (HSP) cell characteristic genes *HSPA1A* and *HSPA18*, cluster 11 cells highly express macrophage characteristic genes *CD68*, *CD163*, and *MS4A7*, and cluster 12 cells highly express eosinophil characteristic genes *CLC* and *OLR1* (**Figure 2E**).

### MC subtypes and their diverse functions in the TME

**Figure 3:**
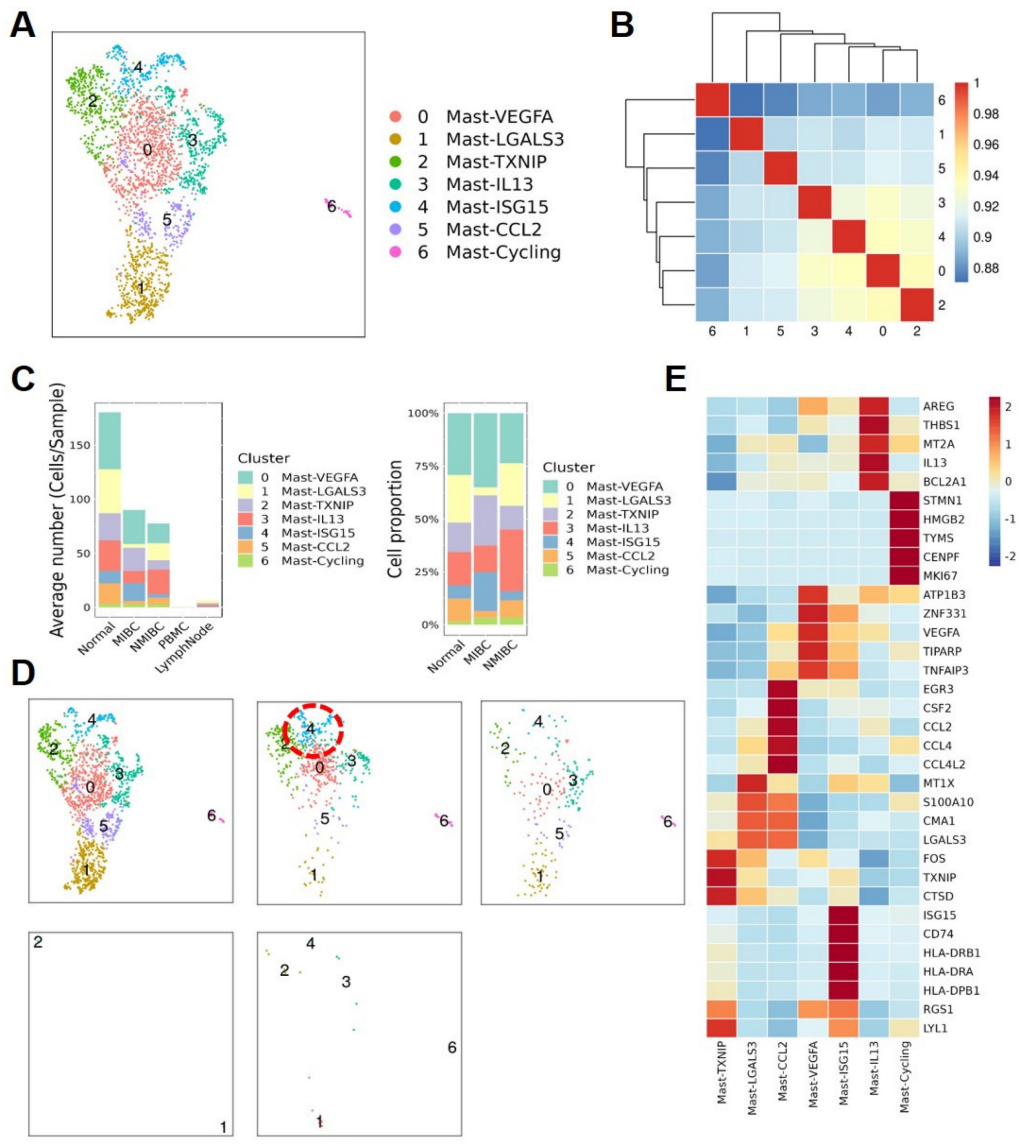
** Functional specialization of the Mast cell subtypes.** (A) The UMAP plot shows that 7 mast cell subgroups are identified after removing mixed cells. (B) Cell subgroup correlation heatmap. (C) Display the average number and cell proportion of mast cell between different subtypes from different sources in human. (D) The UMAP plots show the mast cell subgroups distributions sample sources. (E) Heat map shows the marker gene expression level of mast cell subgroups.

To further describe the diversity of MCs in BCa, we divided these quality-controlled cells into 7 subgroups and named them from subgroup 0 to 6: Mast-VEGFA, Mast-LGALS3, Mast-TXNIP, Mast-IL13, Mast-ISG15, Mast-CCL2, Mast-Cycling (**Figure 3A**). The correlation heatmap shows that these 7 MC subgroups are relatively independent (**Figure 3B**). A bar graph reveals the distribution of MC subgroups in tissues from different sources. We found that infiltrating MCs in MIBC tissues are more abundant than in NMIBC tissues, especially the Mast-ISG15 subgroup (**Figure 3C**). Cell proportion statistics also show that the proportion of the Mast-ISG15 subgroup in MIBC is higher than in other tissues, suggesting that the Mast-ISG15 subgroup may play an important role in the TME of MIBC (**Figure 3D**). The heatmap shows that the Mast-ISG15 subgroup highly expresses interferon-related gene *ISG15* and also high expression of *CD74*, which is involved in MIF-mediated regulation of inflammatory factors (**Figure 3E**). To further understand the function of the Mast-ISG15 subgroup in MIBC tissues, we used the BIOCARTA gene set to enrich the functional analysis of various MC clusters. The results show that the Mast-ISG15 subgroup exhibits strong signals of IFN-α, IL-6, and IFN-γ (**Figure S1**).

### Enrichment of interferon-stimulated transcription factors in MC subgroups

**Figure 4:**
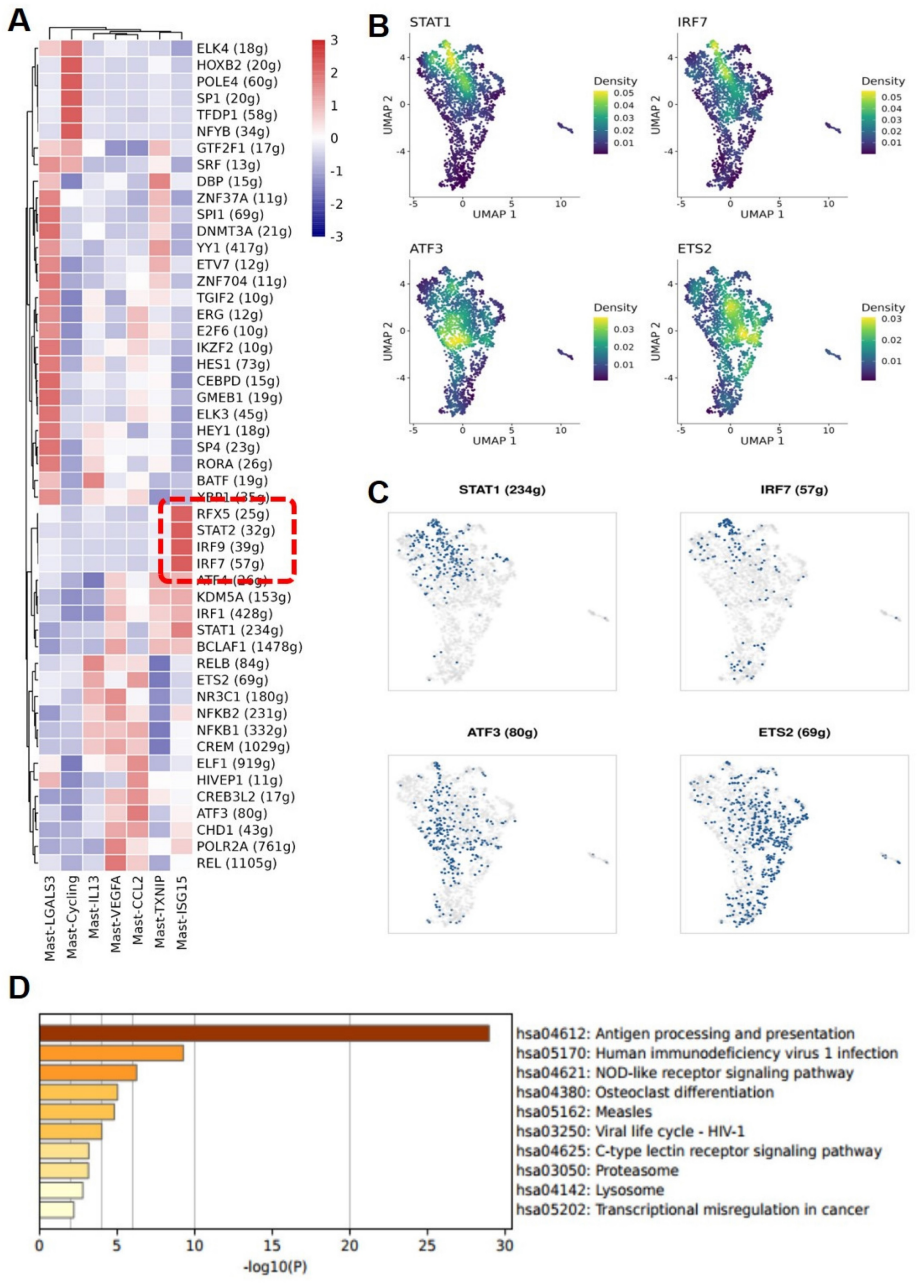
** MAST-ISG15 is an interferon-activated Mast cell subgroup.** (A) Regulon analysis of mast cell subgroups. (B and C) UMAP plot showing the expression pattern of transcript factor STAT1, IRF7, ATF3, and ETS2 and the binarized regulon activity of STAT1, IRF7, ATF3, and ETS2 in mast cells. (D) Functional analysis of Mast ISG15 subgroup by GO Gene Enrichment.

Through further Single-Cell rEgulatory Network Inference and Clustering (SCENIC) analysis of various MC subgroups, it was found that the Mast-ISG15 subgroup indeed exhibited strong transcription factor signals in the interferon pathway, enriching transcription factors STAT1 and interferon regulatory factor IRF7. Additionally, in the NF-κB signaling pathway, it showed strong transcription factor signals, enriching transcription factors NFκB1 and NFκB2 (**Figure 4A, B, C**). Gene Ontology (GO) gene enrichment analysis of the Mast-ISG15 subgroup revealed its association with antigen processing and presentation, as well as the NOD-like receptor signaling pathway (**Figure 4D**). To understand the functions of other MC subgroups, we also conducted GO analysis, showing that the Mast-Cycling subgroup is associated with the cell cycle, DNA replication, and nucleotide metabolism pathways. The Mast-VEGFA subgroup is related to the MAPK signaling pathway, while the Mast-LGALS3 subgroup is associated with oxidative phosphorylation signaling pathway, and it highly expresses the apoptosis-related gene LGALS3. The Mast-TXNIP subgroup is related to apoptosis, and its high expression of the *TXNIP* gene induces cell apoptosis (**Figure S2**).

### Analysis of mast-CCL2 mast cell subgroup in MIBC

**Figure 5:**
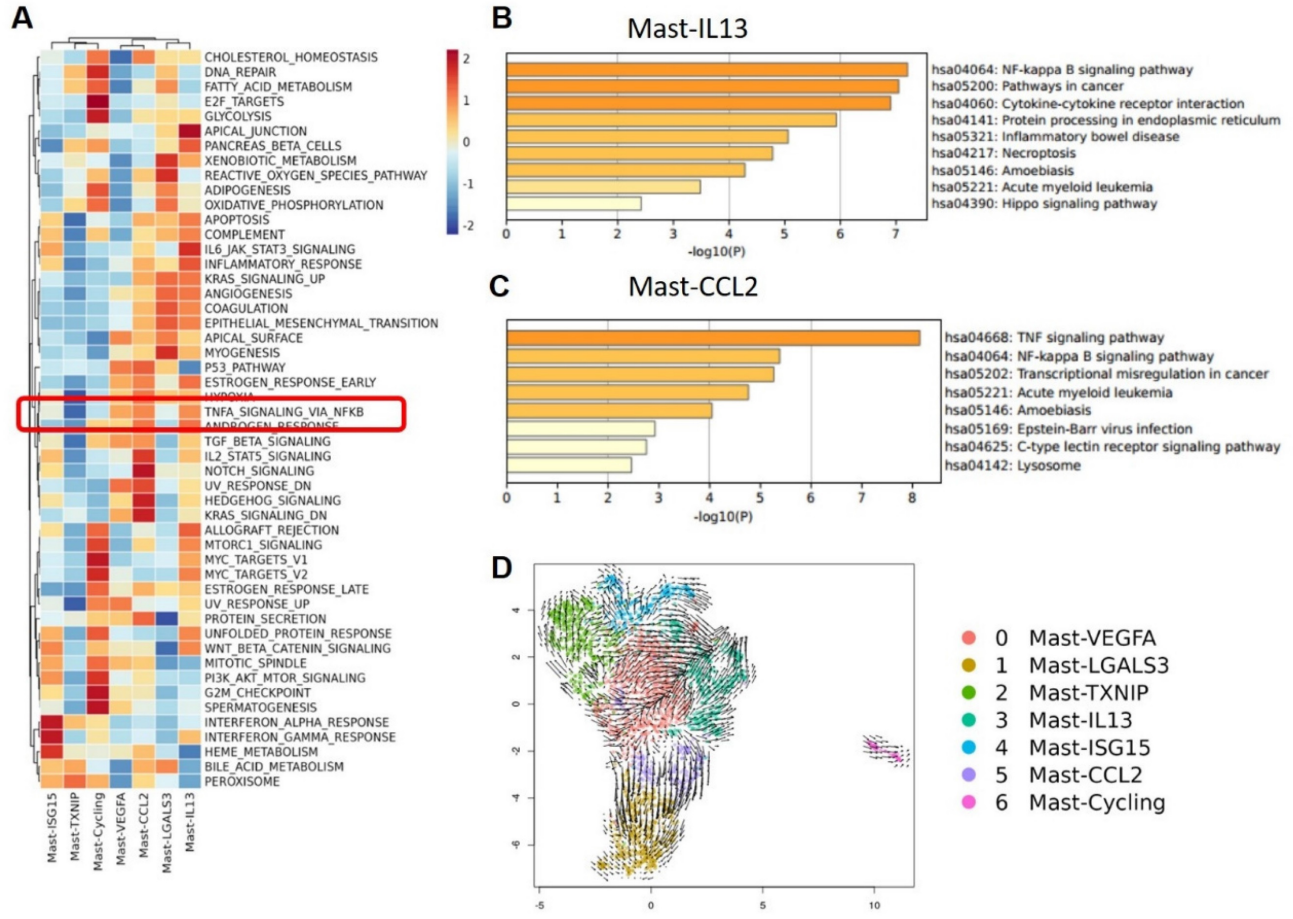
** Functional analysis of Mast-CCL2 and Mast-IL13 subgroups.** (A) Heatmap showing the scaled GSVA scores of hallmark gene sets enriched in mast cell subgroups. (B and C) GO gene enrichment analysis of Mast-CCL2 and Mast-IL13 subgroups shows that their function is related with NF- κB signal pathway. (D) RNA velocity analysis showing the transitions of mast cell subgroups.

Of note is the subgroup Mast-CCL2, which highly expresses CCL2 and CCL4, members of the CCL chemokine family. KEGG gene enrichment analysis of Mast-CCL2 showed a high enrichment in bladder cancer signaling and Toll-like receptor signaling pathways (**Figure S3**). Toll-like receptors (TLRs) are a class of receptors present in natural immune cells such as macrophages and dendritic cells, serving as crucial initiating receptors in activating the classical NF-κB signaling pathway^17^. This subgroup also highly enriches the MAPK signaling pathway and JAK-STAT signaling pathway. The JAK-STAT pathway is a downstream signal of IFN, belonging to a stress-related inflammatory signaling pathway that regulates various biological activities in the body, related to immune responses, immune defense, and can also be involved in processes such as tumor cell survival and immune evasion^18^. Ligands such as IFNs and ILs binding to receptors associated with JAK can activate JAKs, leading to cascade effects and triggering inflammatory responses. To further analyze the functions of various mast cell clusters, we conducted Hallmark gene enrichment analysis, revealing that the Mast-CCL2 subgroup highly enriches the TNF-α-activated NF-κB signaling pathway, suggesting an association between the activation of the Mast-CCL2 subgroup and the NF-κB signaling pathway (**Figure 5A**). Interestingly, in our GO analysis of various MC subgroups, we also found that the activation of the Mast-CCL2 and Mast-IL13 subgroups is related to the NF-κB signaling pathway. Additionally, the Mast-CCL2 subgroup is associated with the tumor necrosis factor (TNF)-α signaling pathway (**Figure 5B, C**).

These results suggest that the Mast-CCL2 subgroup activates the JAK-STAT pathway through IFNs to induce inflammatory responses in the TME. Furthermore, the Mast-CCL2 subgroup may activate the NF-κB signaling pathway through TNF-α, potentially playing a role in the recruitment of peripheral blood monocytes to the tumor by expressing *CCL2* and *CCL4* genes, thereby exerting effects in BCa. Finally, we used RNA velocity analysis to examine the transformation relationships between the various MC subgroups. The results show that a portion of Mast-VEGFA differentiates into the Mast-CCL2 subgroup, and the Mast-CCL2 subgroup may differentiate into the Mast-LGALS3 subgroup (**Figure 5D**), which highly expresses the apoptosis-related gene *LGALS3*. We also observe differentiation from Mast-TXNIP to the Mast-VEGFA and Mast-ISG15 subgroups. It is worth noting that the Mast-IL13 subgroup may originate from the Mast-VEGFA and Mast-ISG15 subgroups. The Mast-VEGFA subgroup highly expresses the gene *TNFAIP3*, which activates the NF-κB pathway, while the Mast-ISG15 subgroup highly expresses the interferon-related gene *ISG15*. This suggests that the activation of the Mast-IL13 subgroup may be related to the IFN signaling pathway or NF-κB signaling pathway. We speculate that in MIBC, the activation of Mast-ISG15 and Mast-VEGFA subgroups leads to the production of numerous inflammatory factors (such as IFN-γ and TNF-α), subsequently activating the Mast-IL13 subgroup to produce IL-13.

### Macrophage cell subtype dynamics and TME differences in MIBC

**Figure 6:**
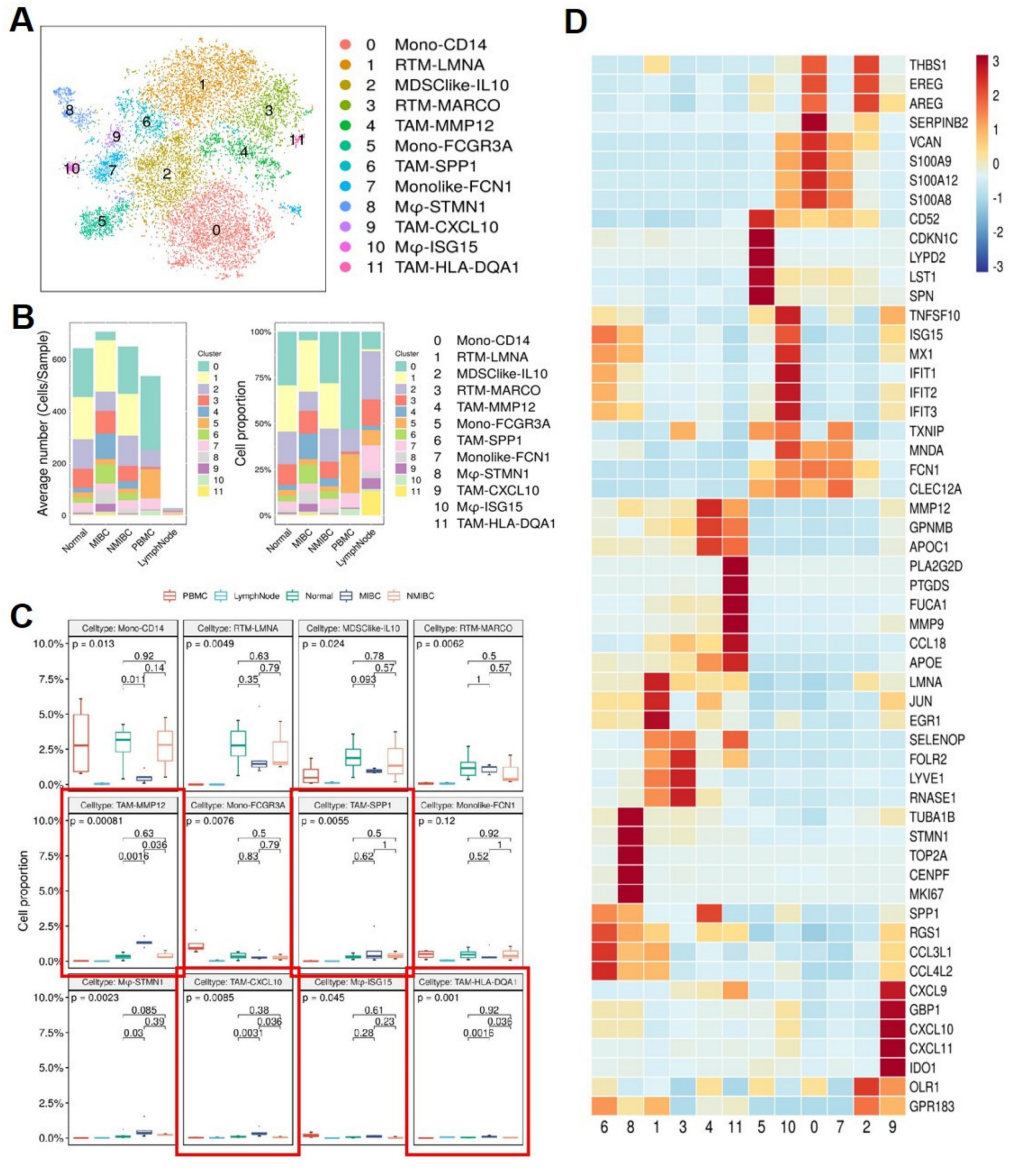
** Mononuclear Phagocyte subgroups and their marker genes.** (A) tSNE plot showing Mononuclear phagocyte subgroups. (B) Average cell numbers and cell proportions of mononuclear phagocyte subgroups in original samples. (C) Boxplot indicated that TAM-MMP12, TAM-SSP1, TAM-CXCL10, and TAM-HLA-DQA1 subgroups increased in MIBC. (D) Heatmap showing the scaled expression levels of mononuclear phagocyte subgroup markers.

To further understand the interaction between MCs and MPs, we reclustered the MP subgroup extracted from the original data. By re-clustering the MP subgroup in BCa, we identified 12 subtypes of MPs (**Figure 6A**), mainly divided into 2 major cell groups. 1 group consists of monocytes, including Mono-CD14, Mono-FCGR3A, and monocyte-like cells Monolike-FCN1. The other group comprises macrophage subgroups, including Mφ-STMN1, Mφ-ISG15, tissue-resident macrophages (RTM-LMNA, RTM-MARCO), tumor-associated macrophages (TAM-MMP12, TAM-SPP1, TAM-CXCL10, TAM-HLA-DQA1), and myeloid-derived suppressor cell-like cells (MDSClike-IL10). Comparing MP subgroups from 5 different tissue samples, it can be observed that monocytes are predominant in the peripheral blood samples, mainly belonging to subgroup Mono-CD14 and subgroup Mono-FCGR3A. Compared to the other 4 tissue samples, the number of monocyte-derived macrophages in lymph nodes is relatively low. Both MIBC and NMIBC exhibit 12 subtypes of MPs, but the quantity and proportion of each cell subtype differ, suggesting differences in bladder cancer progression. In MIBC tissues, tumor-associated macrophages (TAM-MMP12, TAM-SPP1, TAM-CXCL10, TAM-HLA-DQA1) are significantly higher in both cell number and proportion compared to NMIBC tissues and normal tissues (**Figure 6B**). The proportions of different tumor-associated macrophage subgroups vary among different tissues. In MIBC tissues, the proportions of 4 tumor-associated macrophage subgroups (TAM-MMP12, TAM-CXCL10, TAM-HLA-DQA1, TAM-SPP1) are elevated, with TAM-MMP12, TAM-CXCL10, and TAM-HLA-DQA1 subgroups showing significantly higher proportions in MIBC tissues than in NMIBC tissues (*p* <0.05) and adjacent normal tissues (*p* <0.05) (**Figure 6C**). These results suggest that the extensive infiltration of MPs is associated with the occurrence and development of BCa, potentially affecting patient treatment outcomes and prognosis. The differentially expressed gene heatmap shows that within individual MPs subgroups, metalloproteinases (MMPs) and chemokines (CCL, CXCL) are highly expressed, indicating that these MP subgroups may be associated with accelerated tumor progression and impact on disease treatment outcomes (**Figure 6D**).

### Analysis of interaction between MCs and MPs

**Figure 7:**
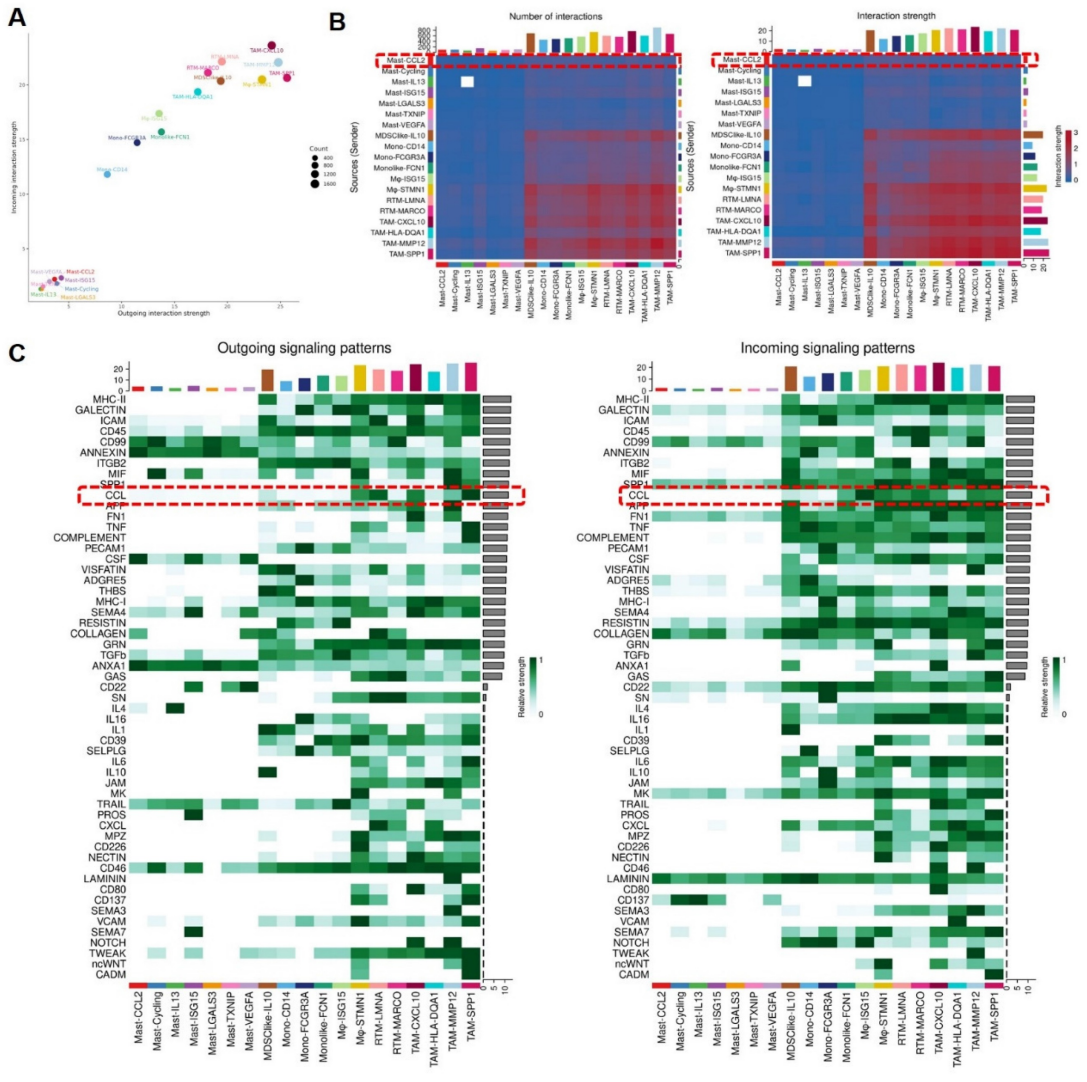
** Mast cells overexpressing CCL2 act on Mononuclear Phagocytes.** (A) Bubble chart infers cell roles. (B) The heat map shows the differences in the number or intensity of interactions between different cell groups. (C) Heatmap showing the contribution of signaling pathways to mononuclear phagocyte subgroups in terms of outgoing or incoming signaling. Mononuclear phagocytes are affected by CCL signaling.

**Figure 8:**
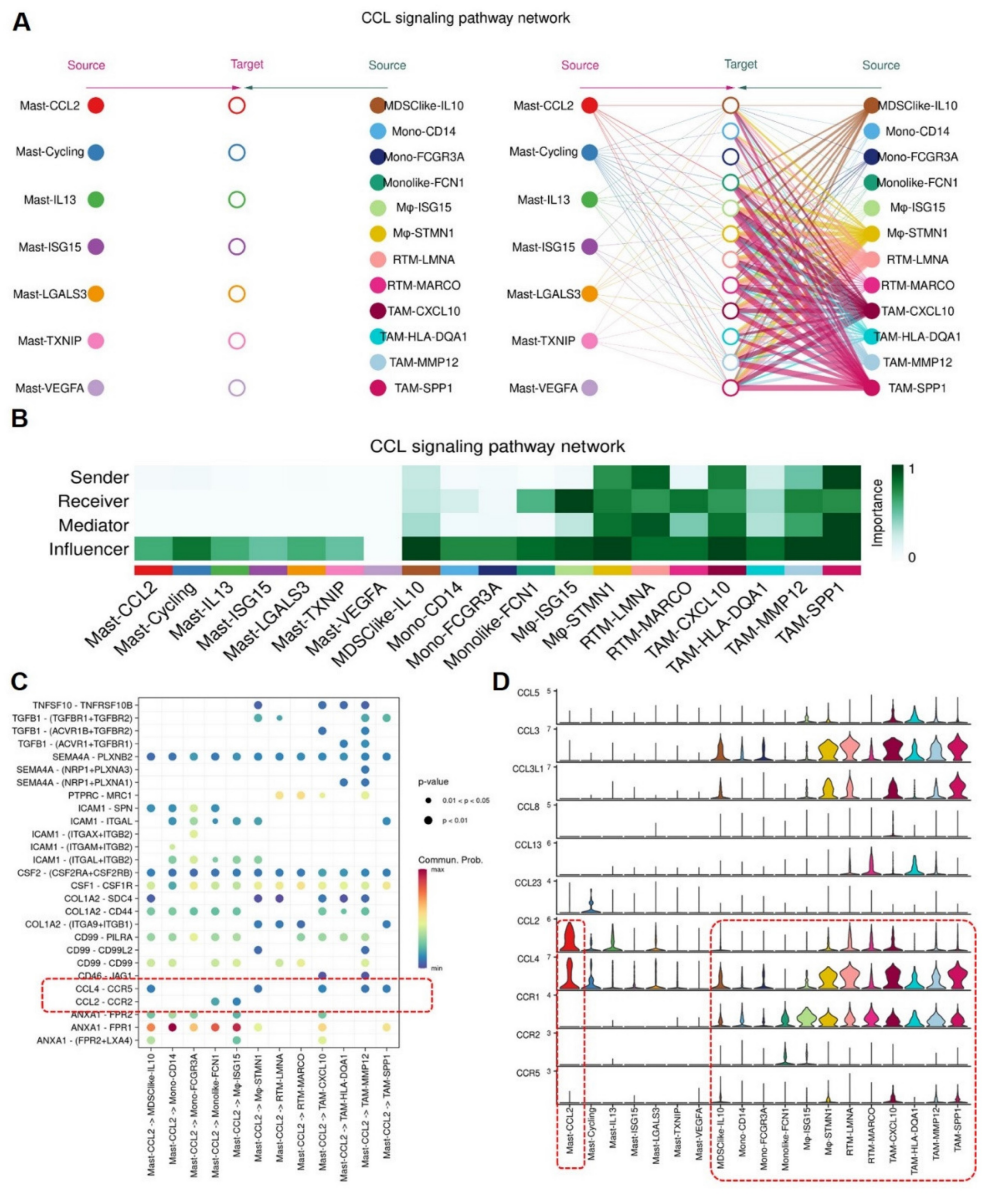
** Mast cells recruit Mononuclear Phagocytes via CCL2-CCR2 ligand-receptor pair.** (A) Mast-CCL2 subgroup interact with mononuclear phagocytes via CCL signaling pathway. (B) Heatmap showing the importance of cell subgroups as senders, receivers, mediators and influencers in CCL signaling pathway. (C) Bubble plot showing cell-cell interactions among mast cell subgroups and mononuclear phagocyte subgroups, and Mast-CCL2 subgroup recruit mononuclear phagocytes through CCL2-CCR2 ligand-receptor pairs. (D) Violin plot presenting the expression levels of CCL signaling pathway genes in mast cell subgroups and mononuclear phagocyte subgroups.

To understand the signal strength of interactions between MCs and MPs subgroups in the TME of BCa, we utilized the CellChat to compare the total number of interactions (receptor-ligand pairs) and interaction strengths (communication intensity differences) of various subgroups. We found that Mast-CCL2 and Mast-ISG15 subgroup exhibit strong signal emission and reception strengths among MC subgroups. The MPs show high numbers and strong intensities of signal emission and reception, such as TAM-CXCL10, TAM-MMP12, and TAM-SPP1 subgroups (**Figure 7A**). Further analysis of the communication process between MCs and MPs as signal senders and the response strength of other subgroups as receivers reveals that when Mast-CCL2 acts as a signal sender, the reaction quantity and intensity of various MP subgroups are strong (**Figure 7B**), suggesting that MCs can attract MPs through CCL2. Subsequently, we analyzed all known signaling pathways from different datasets to compare the effects of signal transmission or reception between MCs and MPs. The results show that both mast cell subgroups and macrophage subgroups (MDSClike-IL10, TAM-SPP1, TAM-CXCL10) can emit CCL signals, while most MP subgroups are influenced by CCL signals (**Figure 7C**). Through the network analysis of the CCL signaling pathway between mast cell clusters and MP clusters, it is revealed that MC clusters can directly interact with MP clusters through CCL ligands, and MP clusters can also influence each other through CCL ligands. Specifically, Mast-CCL2 subgroup directly interacts with MPs (MDSClike-IL10, Monolike-FCN1, Mφ-ISG15, Mφ-STMN1, TAM-CXCL10, TAM-MMP12, and TAM-SPP1) via CCL ligands (**Figure 8A**). By comparing the significant ligand-receptor (L-R) pairs between Mast-CCL2 and various MP subtypes, it is found that Mast-CCL2 acts on MPs through CCL2-CCR2 (Monolike-FCN1, Mφ-ISG15) and CCL4-CCR5 (MDSClike-IL10, Mφ-STMN1, TAM-CXCL10, TAM-MMP12, and TAM-SPP1) (**Figure 8B**). Comparing the distribution of signaling gene expression between MCs and MPs further validates the hypothesis that Mast-CCL2 subgroup may attract MP subgroups through the ligand-receptor pair CCL2-CCR2 (**Figure 8 C, D**). Colony-stimulating factor (CSF) is a cytokine that can stimulate the differentiation of immature bone marrow cells into mature cells and promote the formation of cell colonies in vitro^19^. Among them, macrophage CSF (M-CSF) stimulates the formation of macrophage colonies and promotes granulocyte growth^20^. We analyzed the network effects of the CSF signaling pathway between MC subgroups and MP subgroups. The analysis results show that MC subgroups strongly act on MP subgroups through the CSF ligand (**Figure S4A**). The network centrality analysis of the inferred CSF signaling network reveals that mast cell clusters act as signal emitters through CSF ligand secretion, while MP subgroups act as signal receivers, indicating a potential regulatory role of MC subgroups on MP subgroups (**Figure S4B**).

### Expression of CCL2 and CCR2 in BCa

**Figure 9:**
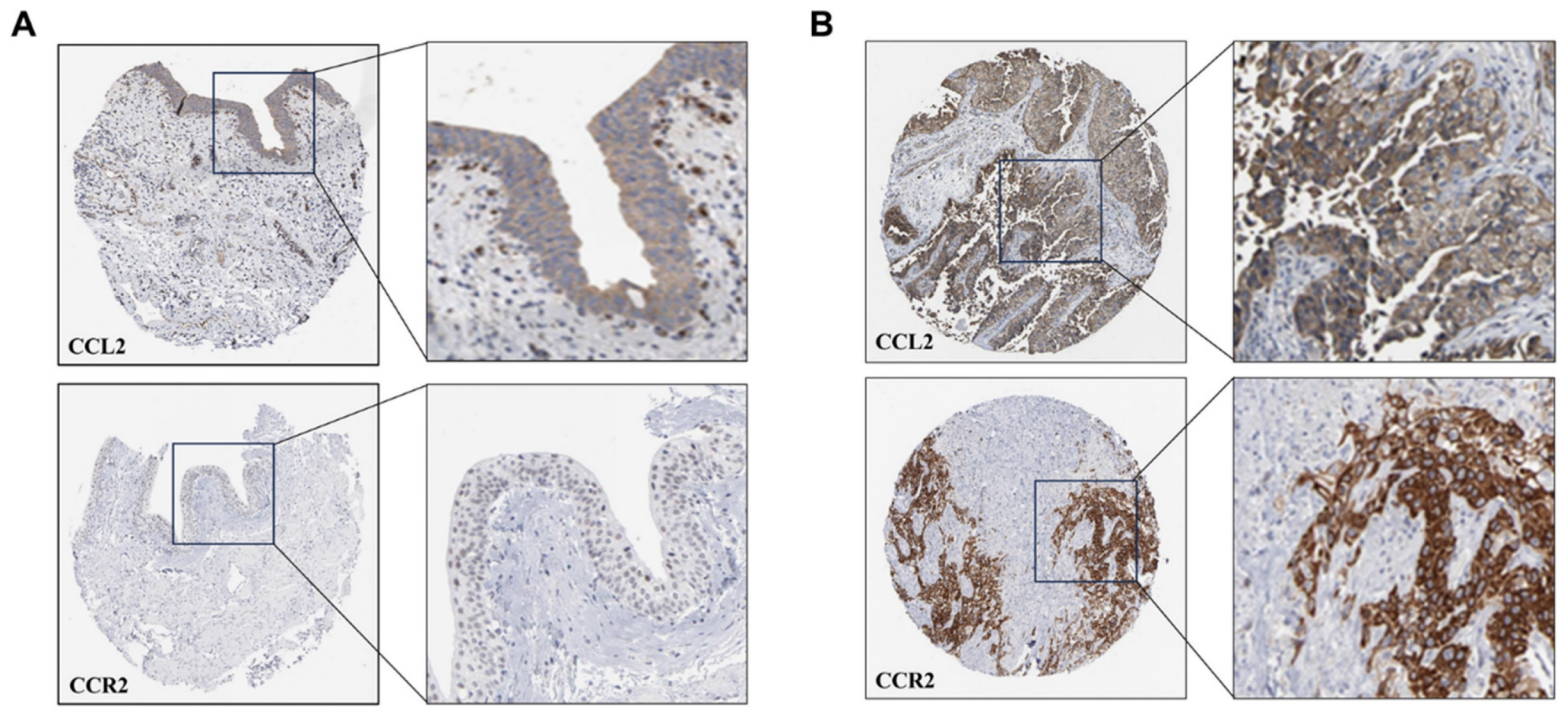
** Expression of CCL2 and CCR2 in Normal Bladder Tissues and Bladder Cancer Tissues.** (A) Tissue immunohistochemical staining of CCL2 and CCR2 in normal bladder tissues: Sourced from the HPA database. (B) Tissue immunohistochemical staining of CCL2 and CCR2 in BCa tissues: Sourced from the HPA database.

To discover the expression of CCL2 and CCR2 in BCa tissues and normal bladder tissues, we retrieved immunohistochemical (IHC) staining data for CCL2 and CCR2 in BCa and normal bladder tissues from the Human Protein Atlas (HPA, https://www.proteinatlas.org) database. It was observed that compared to normal tissues (**Figure 9A**), BCa tissues (**Figure 9B**) exhibited higher infiltration of CCL2 and CCR2. Within the tumor tissues, there was a greater presence of CCL2 and CCR2 in the stroma compared to the cancer nests. These findings suggest that CCL2 and CCR2 play significant roles in the development and progression of BCa.

### IFN-γ induces MCs to secrete CCL2, promoting monocyte infiltration

**Figure 10:**
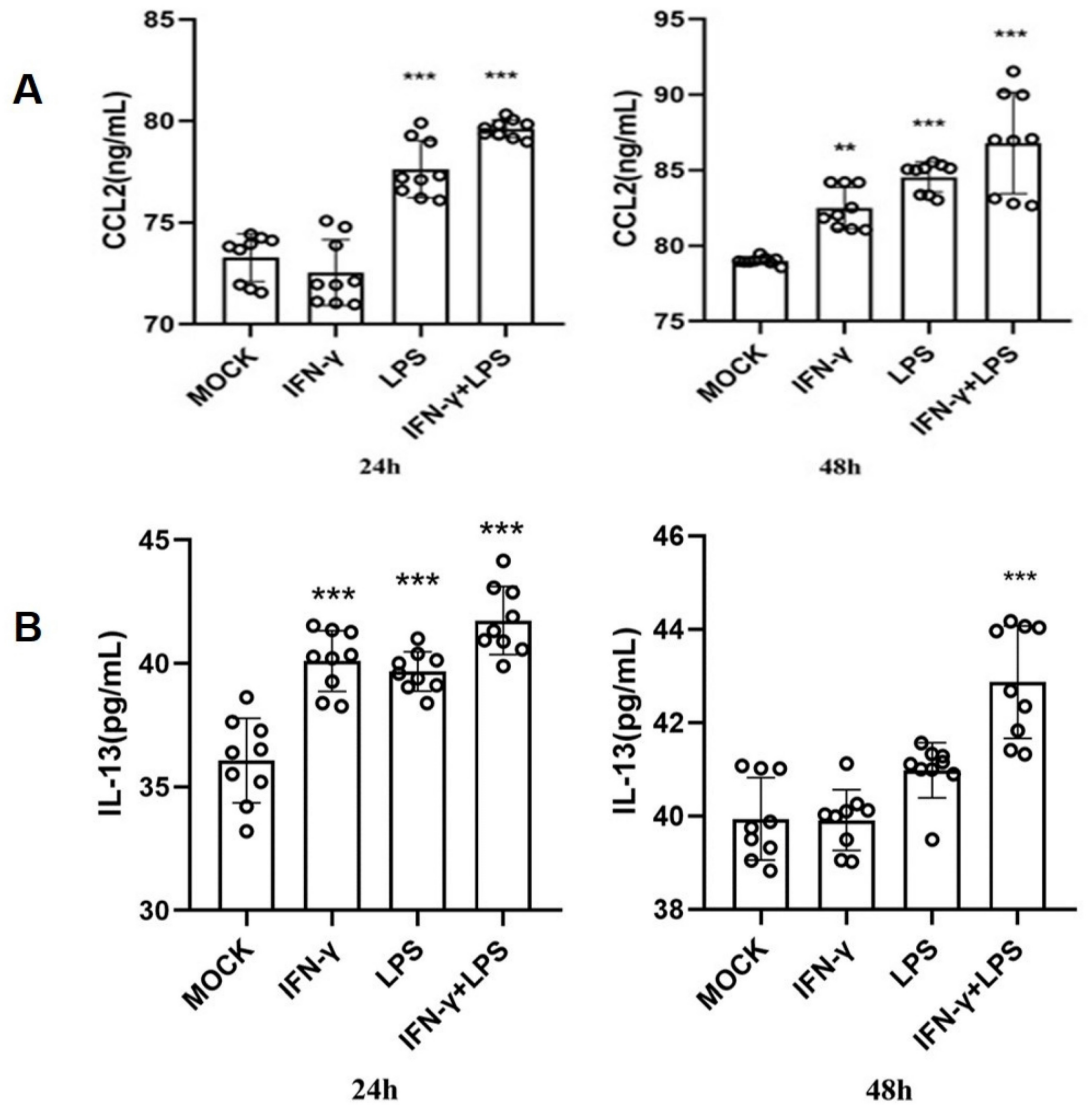
** The CCL2 and IL-13 expression of HMC-1 mast cells with LPS, IFN-γ, and LPS+IFN-γ stimulation are detected by ELISA.** (A) The release of CCL-2 by human HMC-1 mast cells with LPS, IFN-γ, and LPS+IFN-γ stimulation for 24 hours and for 48 hours. (B) The release of IL-13 by human HMC-1 mast cells with LPS, IFN-γ, and LPS+IFN-γ stimulation for 24 hours and for 48 hours. Statistical significance was observed when compared with the MOCK group, indicated as * *p* < 0.05, ** *p* < 0.01, *** *p* < 0.001.

**Figure 11:**
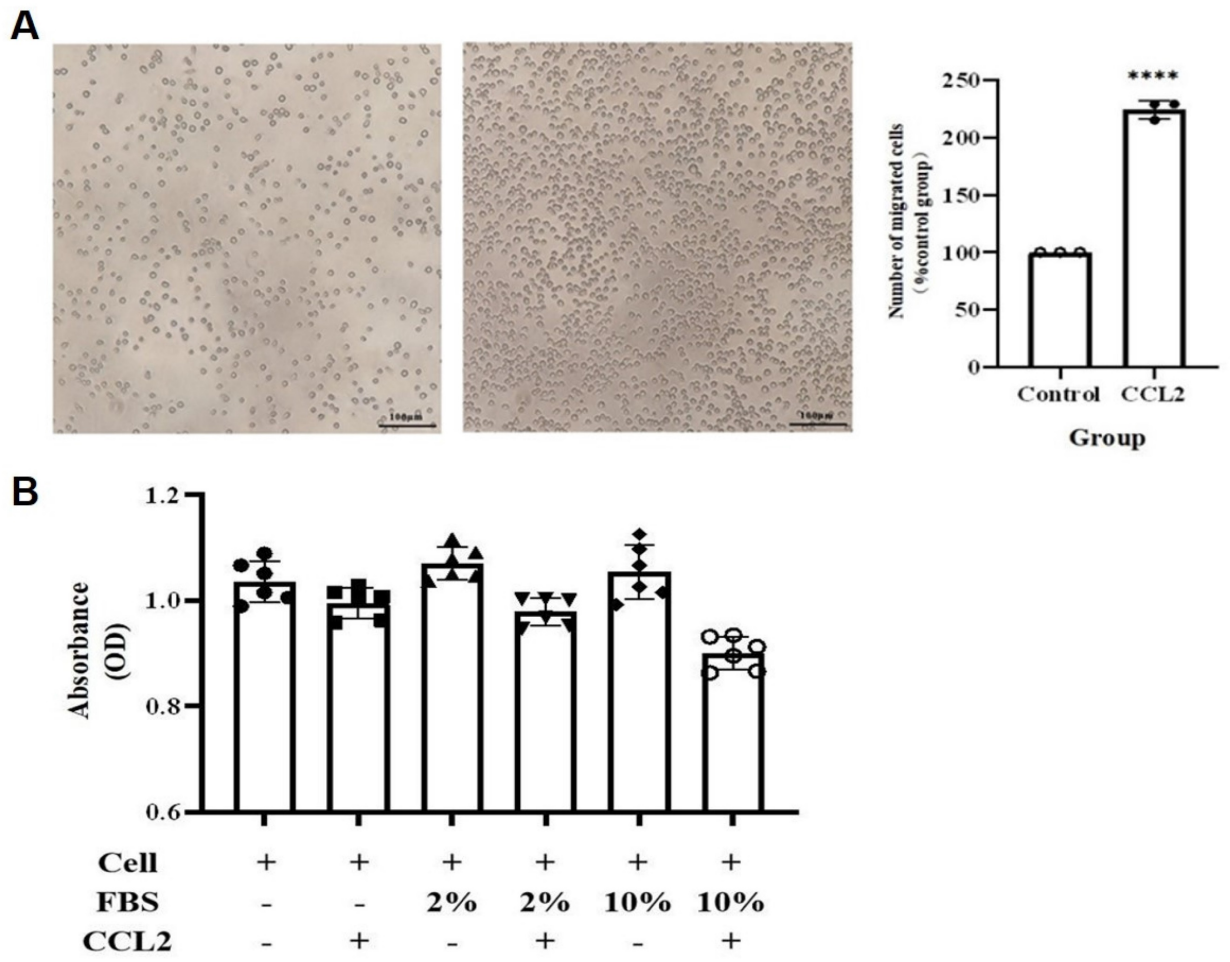
** CCL2 enhances THP-1 monocyte migration without cytotoxicity.** (A) Transwell assay shows CCL2 promotes THP-1 monocyte migration. The scale in the figure is 100 μm; Statistical significance was observed when compared with the MOCK group, indicated as * *p* < 0.05, ** *p* < 0.01, *** *p* < 0.001. (B) CCL2 does not affect THP-1 monocyte proliferation.

To validate the above hypothesis, we cultured the human mast cell line HMC-1 and stimulated the cells with IFN-γ, LPS, or both. We then used ELISA to detect the secretion of IL-13 and CCL2 in the cell culture supernatant. The ELISA results showed that after 24 h of stimulation, compared to the MOCK group, the secretion of CCL2 in the cell culture supernatant significantly increased in the LPS group and the IFN-γ + LPS group (*p*<0.05). After 48 h of stimulation, the secretion of CCL2 in the cell culture supernatant significantly increased in the IFN-γ group, LPS group, and IFN-γ + LPS group (**Figure 10A**). At 24 and 48 h of stimulation, compared to the MOCK group, the secretion of IL-13 in the cell culture supernatant significantly increased in the LPS group, IFN-γ group, and IFN-γ + LPS group (*p*<0.05) (**Figure 10B**). These results suggest that the IFN signaling pathway activated by IFN-γ and the NF-κB signaling pathway activated by LPS can promote MCs to produce the chemokines CCL2 and IL-13. The secretion of IL-13 and CCL2 by MCs is regulated by IFN signaling or NF-κB signaling. To verify that THP-1 monocytes migrate in response to CCL2 stimulation, a Transwell cell migration assay was conducted to compare the number of migrated cells between the group without CCL2 and the group with CCL2. The results showed a higher percentage of cell migration in the group with added CCL2, with a significant statistical difference between the two groups (*p*<0.05) (**Figure 11A**). Finally, to clarify whether serum and CCL2 affect cell proliferation, a CCK-8 assay was performed. The results showed that in the presence of CCL2, cell proliferation did not occur. The results preliminarily demonstrate that under the influence of high serum concentration and CCL2, THP-1 monocytes do not exhibit significant proliferation in the short term (**Figure 11B**).

### Prognostic value of mast cell subtypes in MIBC

**Figure 12:**
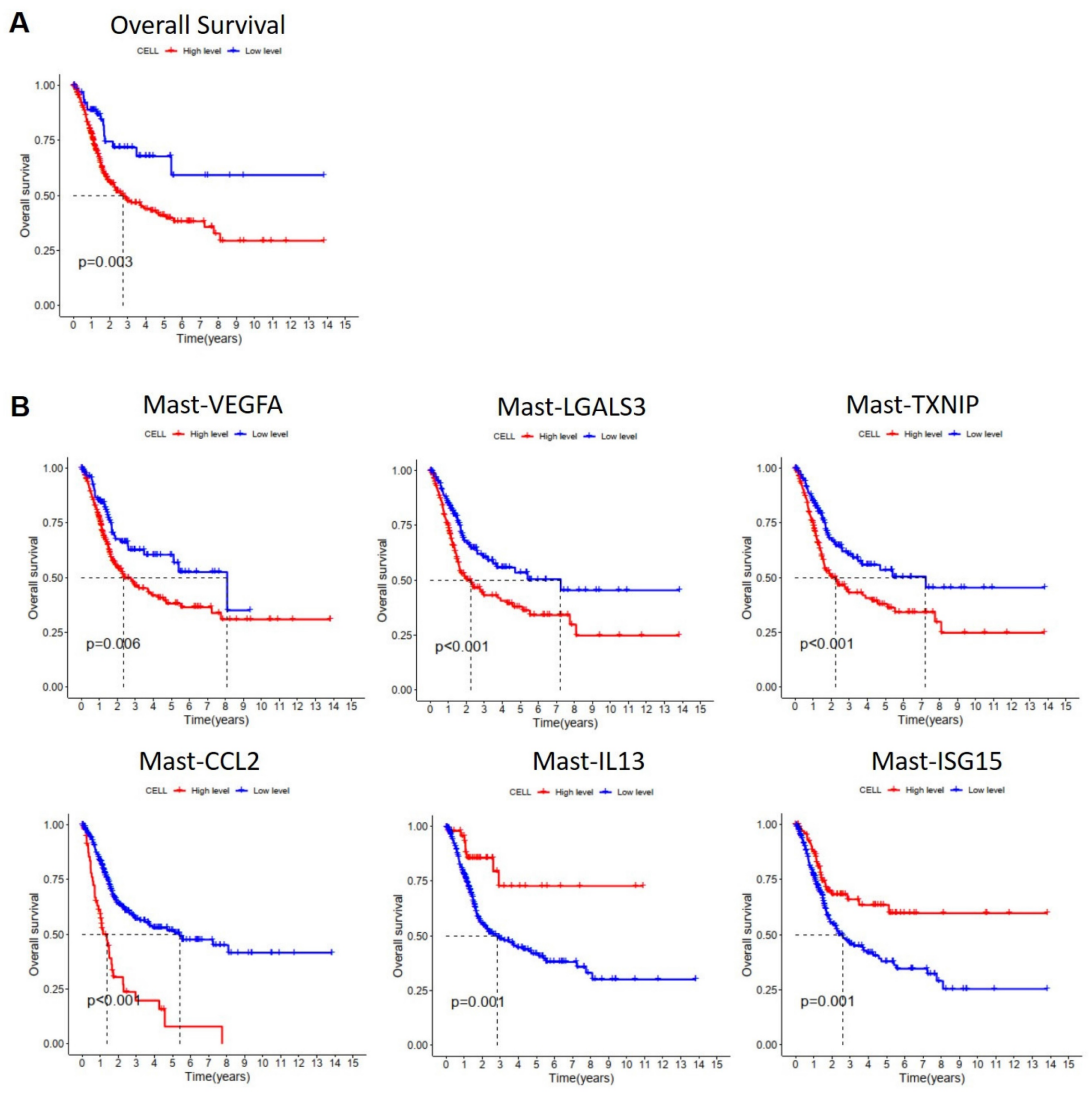
** Effect of mast cells on the survival and prognosis of patients with muscle invasive bladder cancer.** (A) Kaplan-Meier analyzed the effect of mast cell on the survival rate of patients with muscle-invasive bladder cancer (*p* <0.01). (B) Kaplan-Meier analyzed the effect of mast cell subgroups on the survival rate of patients with muscle-invasive bladder cancer (*p* <0.01).

To understand the impact of MCs on the survival prognosis of patients with MIBC, we utilized Lasso regression analysis based on single-cell RNA sequencing results to select characteristic genes for predicting the survival prognosis of MIBC patients with MCs. The Lasso model identified 21 MC genes, of which 15 were considered risk genes and 6 were protective genes (**Figure S4A, B**). Subsequently, we performed Cox regression analysis on these genes, using the TCGA database, and we conducted Kaplan-Meier (KM) analysis on MC infiltration. The results showed that increased MC infiltration was associated with poorer overall survival (OS) in patients with MIBC, indicating a correlation between MC infiltration and adverse prognosis in MIBC patients (**Figure 12A**). To investigate the impact of various MC subgroups on the survival prognosis of patients with MIBC, we utilized the TCGA database in combination with characteristic gene sets of each MC subgroups for KM analysis. The results revealed that increased infiltration of Mast-VEGFA, Mast-LGALS3, Mast-TXNIP, and Mast-CCL2 was associated with shorter overall survival in patients with MIBC, indicating a correlation between these MC subgroups and adverse prognosis (*p* < 0.05). Moreover, increased infiltration of Mast-IL13 and Mast-ISG15 subgroups were associated with better OS in patients with MIBC, suggesting a correlation between these MC subgroups and favorable prognosis (*p* <0.05) (**Figure 12B**). These findings indicate the heterogeneity of the effects of various MC subgroups on BCa, highlighting MCs as potential therapeutic targets for MIBC.

## Discussion

The TME plays a crucial role in the progression of cancer, and the changes in tumor development are closely related to the status of the TME^21^. A comprehensive understanding of the various components in the TME is essential for identifying new therapeutic strategies for BCa. With the advancement of single-cell transcriptome sequencing technology, the composition, heterogeneity, and interactions of immune cells in the tumor microenvironment are gradually being understood. Numerous studies have attempted to elucidate the role of Tumor-Associated Mast Cells (TAMCs) in tumor development and progression. In most tumors, TAMCs exhibit functional heterogeneity, either promoting or inhibiting tumor growth, or in some cases, having no impact on tumor growth. Studies have reported high levels of MC infiltration in the tumor microenvironment of various cancers, such as gastric cancer and thyroid cancer^22^-^25^. In the TME, the interaction between MCs and tumor cells may lead to the activation of MCs, inducing the release of active mediators. The released tryptase from MCs may promote angiogenesis by activating matrix metalloproteinases. Tryptase plays an important role in degrading the extracellular matrix and releasing angiogenic factors. MCs may also be associated with cellular tolerance, as they exert immunosuppressive effects by releasing the immunosuppressive factor IL-10. MCs play diverse roles in the tumor microenvironment, indicating their heterogeneity. However, the specific role of tumor-associated mast cells in the bladder cancer tumor microenvironment is not yet fully understood.

In this study, our sc-RNA sequencing data identified 7 subtypes of MCs. The discovery of various MC subgroups suggests that MCs play different roles in the BCa TME. The interferon-related MC subgroup Mast-ISG15 has the highest proportion among the infiltrating MC subgroups in MIBC. The Mast-ISG15 subgroups highly expresses interferon-related gene *ISG15*, as well as NF-κB signaling pathway transcription factors *NFκB1* and *NFκB2*, indicating that IFN signaling is a significant feature in the microenvironment of MIBC. Interferons are important immune system components that regulate tumor development^26^. Interferons have dual roles in immune suppression and promoting tumor development; they also play a crucial role in tumor immune surveillance by promoting the transformation of M1 macrophages and inhibiting the function of regulatory T cells^27^, ^28^. The dual immunomodulatory effects of interferons may impact the balance between tumor immune clearance and immune escape^29^. Chemokines regulate the activation, recruitment, phenotype, and function of immune cells during tumor development, contributing to the interactions between immune cells in the TME^30^. The results of this study show the presence of a Mast-CCL2 subgroup with high expression of the chemokine genes CCL2 and CCL4 in MIBC. This subgroup is highly enriched in the TNF-α-activated NF-κB signaling pathway and the JAK-STAT signaling pathway. Additionally, this subgroup is highly enriched in bladder cancer signaling and Toll-like receptor signaling pathways. Toll-like receptors are crucial initiating receptors in activating the classical NF-κB signaling pathway. These results suggest that the Mast-CCL2 subgroup may initiate the JAK-STAT pathway through interferon activation in the TME, leading to inflammation. Furthermore, Mast-CCL2 cells activate the NF-κB signaling pathway through TNF-α, producing the chemokines CCL2 and CCL4. CCL2 and CCL4 can recruit peripheral blood monocytes to the TME, thereby playing a role in MIBC.

We continued to analyze single-cell RNA sequencing data and identified 12 subgroups of MP. Tumor-associated macrophages (TAM-MMP12, TAM-SPP1, TAM-CXCL10, TAM-HLA-DQA1) infiltrate significantly higher in MIBC tissues compared to NMIBC tissues and adjacent normal tissues, indicating that tumor-associated macrophages play an important role in the TME of MIBC and further research is needed to understand how these macrophages reach the tumor site. SPP1+ TAM was initially discovered by Zhang et al. in single-cell RNA sequencing studies of colon cancer^31^. This subgroup highly expresses genes such as SPP1, MARCO, VEGFA, and SDC2. SDC2 on this subgroup can interact with MMP2 on tumor-associated fibroblasts and endothelial cells, promoting tumor growth and metastasis^32^. A pan-cancer scRNA-seq study on 15 common tumors revealed the presence of SPP1+ TAM in breast cancer, pancreatic cancer, lung cancer, colon cancer, endometrial cancer, nasopharyngeal cancer, ovarian cancer, and thyroid cancer tissues. At the transcriptome level, this subgroup co-expresses characteristic genes of both M1 and M2 type TAMs, with a predominance of M2 type TAM genes and high expression of angiogenesis-related genes^33^. Understanding how a large number of macrophages infiltrate the tumor microenvironment requires further investigation. MC play a functional role in the TME by interacting with other immune cells^34^. During tumor proliferation and metastasis, mediators released by MCs in the TME, such as NGF, PDGF, VEGF, IL-8, and IL-10, can induce macrophages to polarize towards an M2-like phenotype, promoting immune suppression, angiogenesis, tumor cell extravasation, and metastasis^35^, ^36^. Cell interaction analysis shows that the Mast-CCL2 subgroup signals to MP subgroups, influencing MDSClike-IL10, TAM-SPP1, and TAM-CXCL10 subgroups. Furthermore, Mast-CCL2 acts on MP subgroups through CCL2-CCR2 signaling. Previous research reports that CCL2 has chemotactic activity on monocytes and eosinophils, making it a key chemokine for regulating monocyte/macrophage migration and infiltration^36^. Our study suggests that MCs may attract MPs to the TME by secreting CCL2.

To further validate the functions of Mast-IL13 and Mast-CCL2 subgroups at the protein level under the regulation of IFN and NF-κB signaling pathways, a series of validate experiments were designed and conducted. IFN-γ treatment of MCs directly activates the IFN signal. LPS, also known as lipopolysaccharide, a unique chemical component in the outer membrane of G- bacteria, can bind to TLR4 on the cell surface, activating downstream NF-κB signaling^38^. Results indicate that under the action of IFN-γ or LPS, MCs enhance the secretion of the cytokine CCL2 and IL-13, confirming the regulation of the Mast-IL13 and Mast-CCL2 subgroup by IFN and NF-κB signals. The presence of Mast-IL13 and Mast-CCL2 subgroups reflects the strong IFN signaling in BCa, especially in the TME of MIBC. IFN-γ is mainly secreted by activated CD8+ T cells, consistent with our previous findings of a significant increase in CD8+ T cell numbers and proportions in the TME of MIBC^39^. Transwell results suggest that CCL2 in the BCa TME has a chemotactic effect, and Mast-CCL2 can recruit peripheral blood monocyte-derived macrophages to the tumor microenvironment by secreting the chemokine CCL2.

Finally, we found that MC infiltration is associated with poor prognosis in patients with MIBC. Further research on the impact of various MC subgroups on the prognosis of patients with MIBC revealed that the more infiltration of Mast-VEGFA, Mast-LGALS3, Mast-TXNIP, and Mast-CCL2, the worse the overall survival of patients with MIBC, correlating with poor prognosis. This indicates the heterogeneity of the effects of various MC subgroups on BCa, reflecting the complexity of cells in the TME.

## Conclusion

This study utilized cutting-edge single-cell RNA sequencing technology to systematically characterize the MCs landscape in the BCa TME. Experimental validation showed that under the regulation of IFN or NF-κB signaling, MCs recruit MPs through CCL2 chemotaxis. Our study provides a theoretical basis for further elucidating the role of MCs in the development of BCa, clarifying the immune mechanisms of MCs and improving BCa treatment outcomes.

## Supplementary Material

Supplementary figures.

Supplementary table 1.
