# Supplementary material for: Single cell RNA-Sequencing Reveals Mast Cells Enhance Mononuclear Phagocytes Infiltration in Bladder Cancer Microenvironment
Source: J Cancer. 2024 Sep 3;15(17):5672–90. doi: 10.7150/jca.99554 (PMC11414625; doi:10.7150/jca.99554)
Supplement: Supplementary file 1 — Supplementary figures. [file jcav15p5672s1.pdf]

Supplementary materials

Supplemental file 1: Table S1. Patient information.

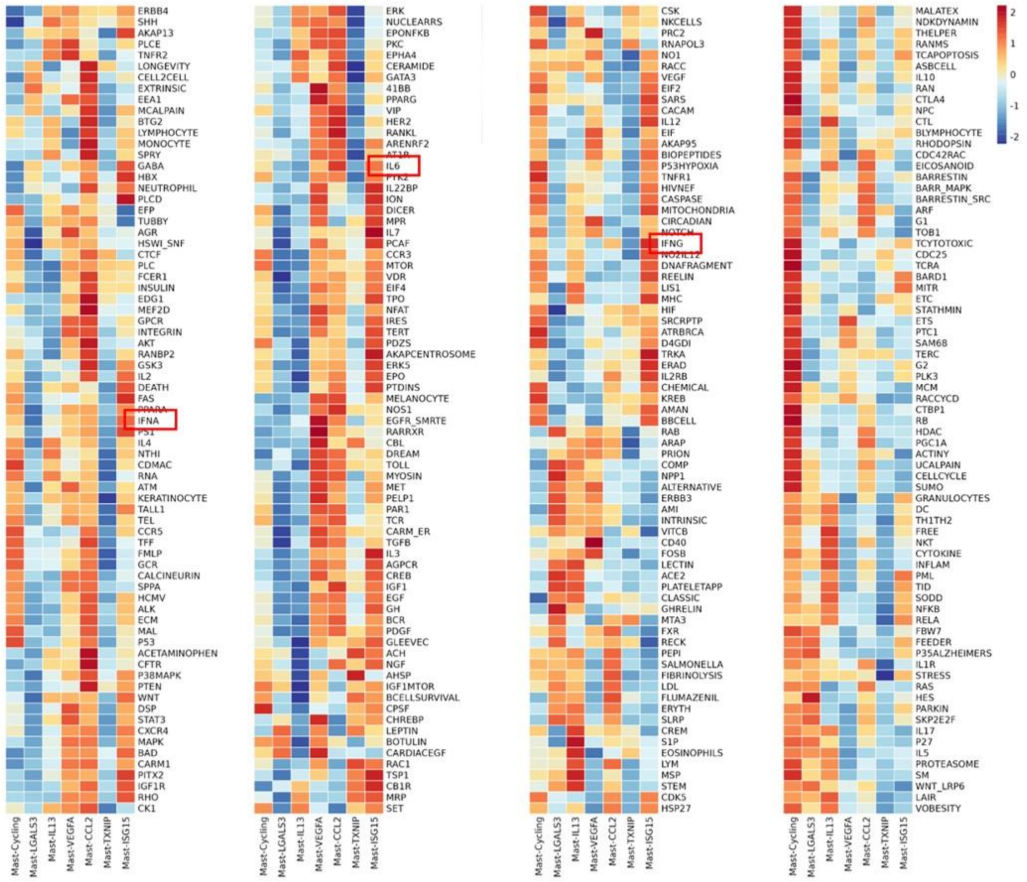

Figure S1. Heatmap showing the scaled GSVA scores of BioCarta gene sets enriched in mast cell subgroups.

As BioCarta gene sets contained 217 gene sets, the experimental results are divided into four parts for presentation.

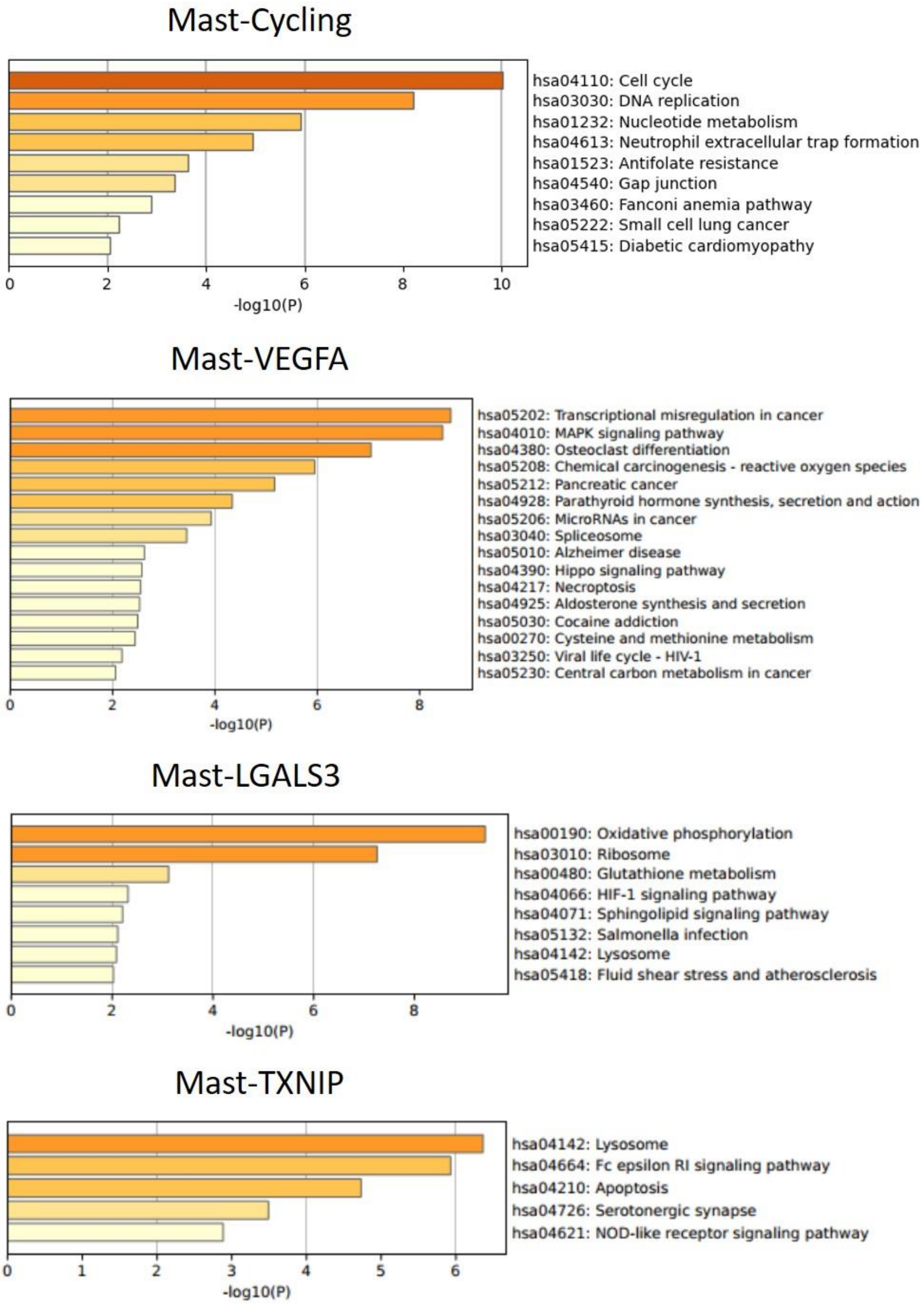

Figure S2. Functional analysis of mast cell subgroups Mast-VEGFA、Mast-LGALS3、Mast-TXNIP、Mast-Cycling by GO Gene Enrichment.

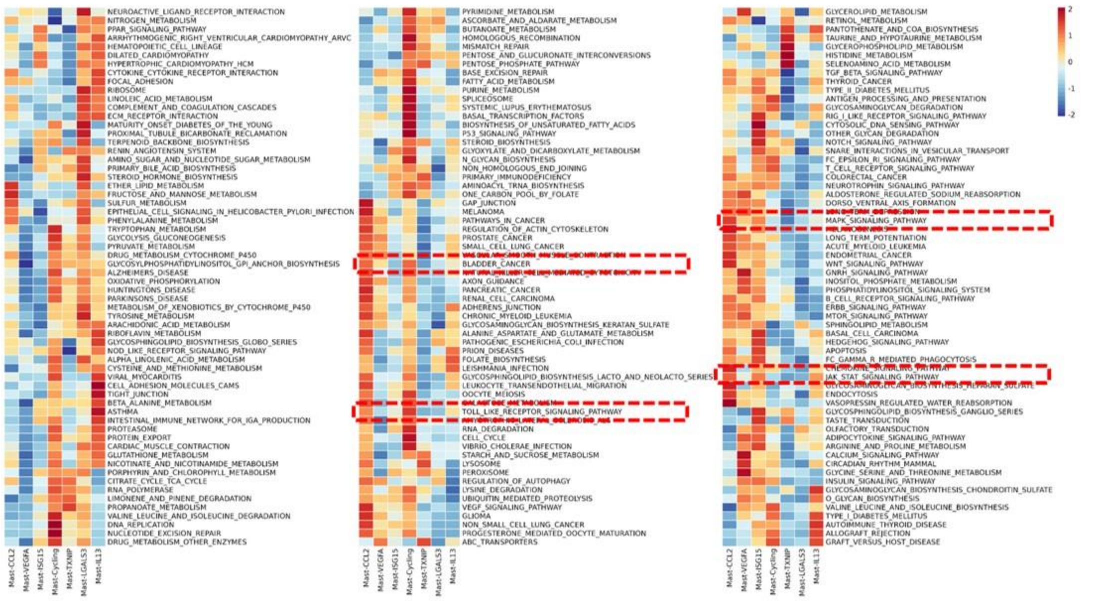

Figure S3. Heatmap showing the scaled GSVA scores of KEGG gene sets enriched in mast cell subgroups

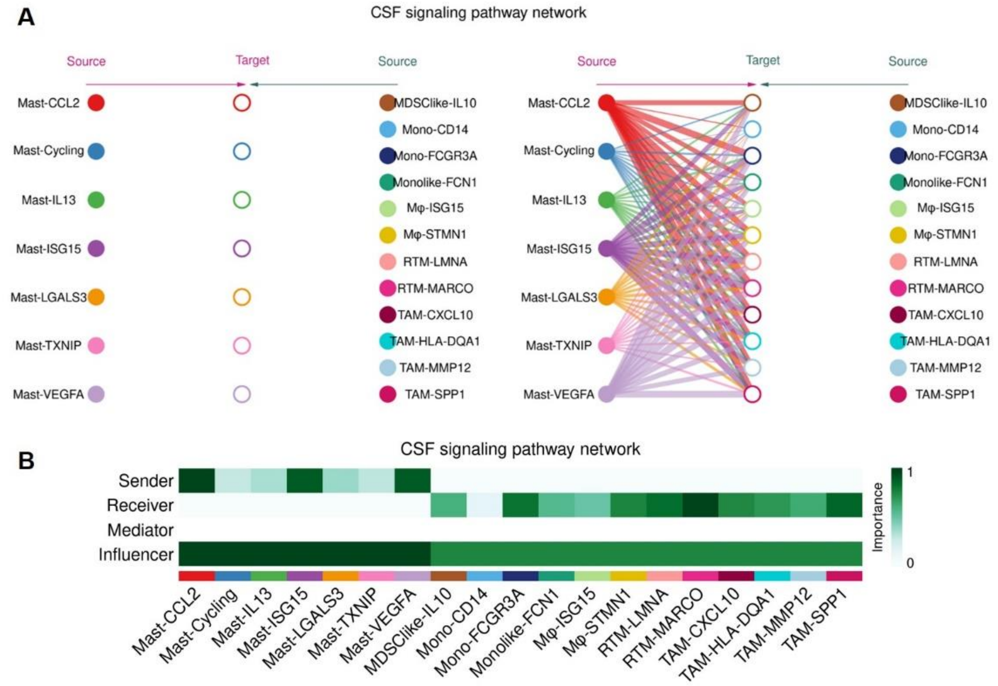

Figure S4. Mast-CCL2 affects Mononuclear Phagocytes through CSF signaling

(A) Mast cell subgroups interact with mononuclear phagocytes via CSF signaling pathway.

(B) Heatmap showing the importance of cell subgroups as senders, receivers, mediators and influencers in CSF signaling pathway.

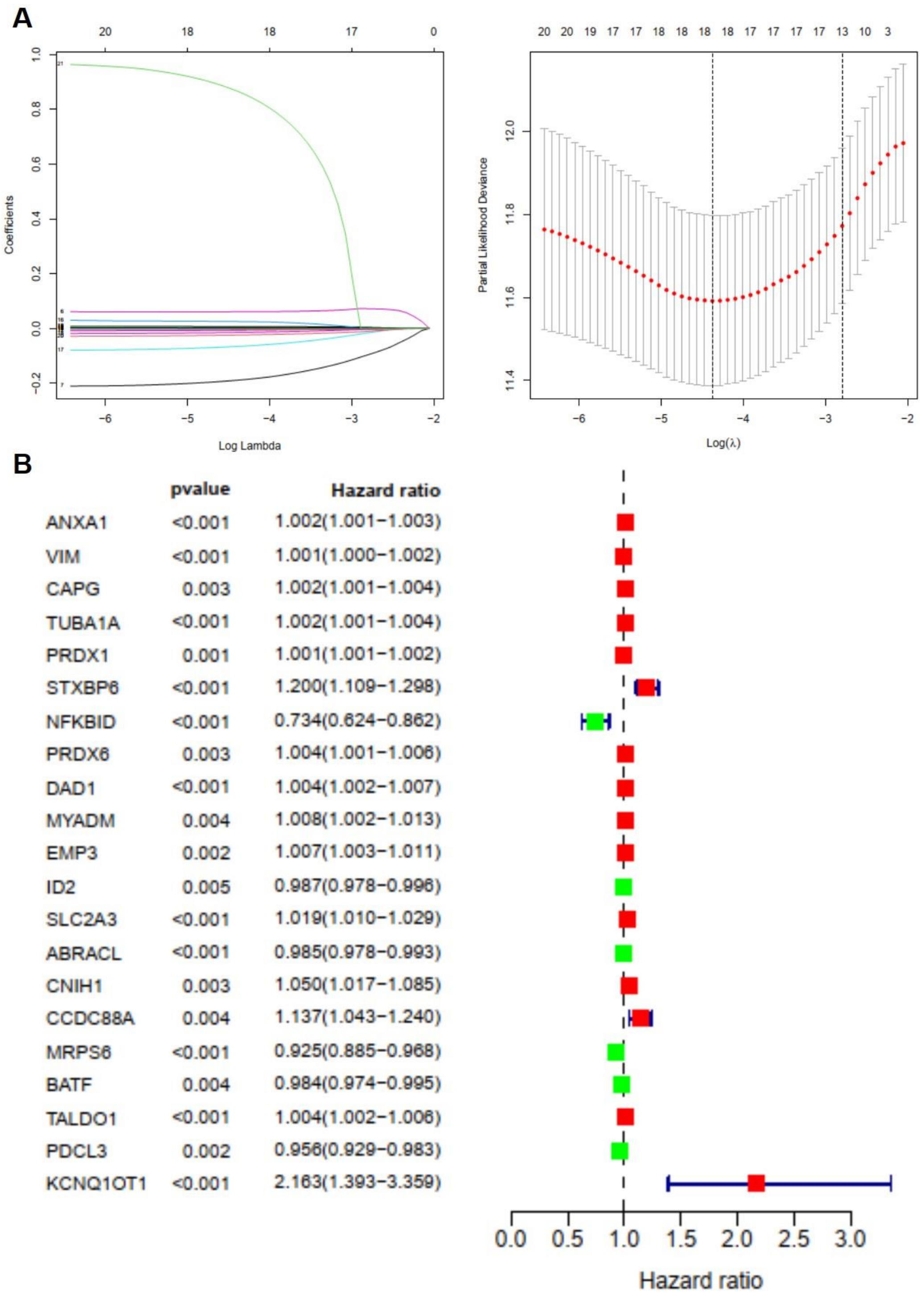

Figure S5. To establish a feature gene Lasso model for predicting the survival prognosis of patients with muscle invasive bladder cancer

(A) A Lasso coefficient path map of 21 prognostic characteristic genes of mast cell based on single cell RNA sequencing data.

(B) Cross validation of Lasso regression Curve Forest map: Cox regression analysis of 21 mast cell characteristic genes.
